# Supplementary material for: Are carbon emissions trading and green financial instruments synergistic? -Comprehensive quantitative research based on content analysis
Source: PLoS One. 2024 Mar 7;19(3):e0298601. doi: 10.1371/journal.pone.0298601 (PMC10919601; doi:10.1371/journal.pone.0298601)
Supplement: S1 Data — (ZIP) [file pone.0298601.s001.zip › Data/地级市Z_F工作报告.zip!/╣π╬≈/╟╒╓▌╩╨/└┤╫╘╣1⁄2╓┌║┼í╛┬φ┐╦╩2╛▌═°í┐_2017.docx]

各位代表：

　　现在，我代表市人民政府向大会报告工作，请予以审议，并请各位政协委员和其他列席会议的同志提出意见。

　　一、2016年工作回顾

　　过去一年，在党中央、国务院和自治区党委、政府及市委的坚强领导下，全市上下按照“五位一体”总体布局和“四个全面”战略布局的要求，认真贯彻落实五大发展理念，牢牢把握稳中求进工作总基调，大力实施“开放创新、港城联动、产业强市、生态惠民”发展战略，扎实做好稳增长、促改革、调结构、惠民生、防风险等各项工作，实现了“十三五”良好开局。

　　（一）着力稳增长，主要指标增速位居前列

　　紧抓发展第一要务不动摇，精准发力，打好“六稳”组合拳。及时出台实施降成本35条政策措施，开展“非公企业接待日”活动，帮助企业解决用电、资金、物流等生产运营难题，鼓励大众创业、万众创新。组织实施“两个年”活动，统筹抓好540个重点项目和124个园区基础设施项目，总投资500万元以上项目竣工2568个、同比增长41.5%。开展精准大招商，引进到位资金522亿元。稳增长工作在国务院第三次大督查中得到通报表扬，贯彻落实中央和自治区政策措施成效得到自治区政府激励表扬，多项主要经济指标增速位居全区前列。2016年地区生产总值完成1102亿元，增长9%；规模以上工业总产值1524.1亿元，增长11.7%；固定资产投资950.9亿元，增长17.4%；财政收入154.08亿元，负增长5%；港口货物吞吐量6953.5万吨，其中集装箱吞吐量137.4万标箱、增长45.9%；社会消费品零售总额373.6亿元，增长12%；城镇和农村居民人均可支配收入分别增长7.3%和9.3%，城镇登记失业率3.14%。

　　（二）着力调结构，产业体系发生积极变化

　　千方百计培育发展千百亿元产业、做大做强县域工业、提升发展现代服务业，三次产业比例优化调整为20.1∶43.7∶36.2，第二产业增加值占比提高3.3个百分点。临港产业加快集聚。石化产业园总体规划完成修编，玉柴石化30万吨异辛烷等项目开工建设，华谊煤基多联产等项目前期工作加快推进；中船修船资源整合项目试产，力顺轻型载货汽车改造升级项目建成，千亿元石化、装备制造业取得新进展。国投电厂二期、鲁花食用油项目竣工投产，中粮、金桂等企业增产增效，能源、粮油食品加工、造纸等百亿元产业发展壮大。大力发展高新技术产业，启动创建国家级高新区，卓能新能源成为广西首家列入国家《汽车动力蓄电池行业规范条件》目录的企业，引进光电产业基地等一批项目，新增高新技术企业6家、总数达42家。海洋工程、海洋生物医药等海洋新兴产业加快发展，形成经济新增长点。全市新增规模以上工业企业16家，县域规模以上工业总产值突破1000亿元、达1067亿元。提速发展现代服务业，保税港区跨境电商进口业务正式启动，农村众包物流+电商综合服务平台建成运营，灵山县获批为全国电子商务进农村综合示范县，钦南区成功创建广西特色旅游名县并列入全国全域旅游示范区创建名单，全市接待游客和旅游收入分别突破1800万人次和170亿元；远景资源公司在“新三板”挂牌，金融业健康稳定发展。着力推进农业现代化，粮食产量和面积连续六年超额完成自治区任务，新建农业部园艺作物标准化生产基地4个、自治区级现代特色农业（核心）示范区3个、自治区休闲农业与乡村旅游示范点4个，新增广西特色富硒农产品品牌3个、农民专业合作社667家、家庭农场83家，新建“双高”糖料蔗基地1.5万亩，农林牧渔业投资增长47.9%，总产值增长4.2%。水产畜牧兽医工作获农业部表彰，“钦州大蚝”成为广西养殖产品类唯一入选2016年度“中国品牌价值评价榜”的品牌，品牌价值45.48亿元。

　　（三）着力扩开放，钦州平台凸显集聚效应

　　坚持内外联动，培育发展新动能、新优势。夯实做强钦州平台。中马钦州产业园区进入项目建设收获期，稀土高铁铝合金电缆研发生产基地一期、中马国际科技园等启动建设，慧宝源项目试投产，国家级燕窝及营养保健食品检测重点实验室竣工使用；新引进入园项目40个，总投资240亿元。全力支持钦州保税港区加快发展，整车进口、国际商品直销等特色业务快速拓展，肉类指定口岸进口等业务正式开展，进境水果指定口岸通过国家验收，直升飞机临时起降业务获批筹建营运，柳州汽车“南车北运”海上新通道正式打通，全年进口酒类207万升、汽车3100辆。中国—东盟港口城市合作网络取得实质进展，中方秘书处正式挂牌，与老挝琅勃拉邦港缔结为国际姐妹港，中国—东盟港口物流信息中心正式启用，在马来西亚关丹市成功举办首届“钦州日”交流活动。北部湾集装箱干线港地位进一步确立。启动10万吨级集装箱双向航道扩建、集装箱货运站等项目，国投煤炭码头、30万吨级支航道、金鼓江航道等项目竣工。试运营贵阳至钦州港货运专列，新开通至中东地区、马来西亚等5条外贸集装箱航线，面向东盟、辐射腹地的物流大通道更加便捷，港口物流逆势攀高，外贸集装箱吞吐量增长199.1%。探索先行先试开放政策。开展自由贸易试验区8个方面48条政策先行先试，电子口岸一期投入运营，成功办理广西首单“单一窗口”业务；cepa服务贸易协议先行先试正式启动。钦州港口岸联检大楼、果子山边地贸口岸联检楼建成。加工贸易进出口额28.3亿美元，钦州跻身全国外贸50强城市，金桂浆纸“金蝶兰”获评广西出口名牌。

　　（四）着力促创新，体制机制焕发生机活力

　　坚持改革创新，增强发展内生动力，提升竞争优势。全力推进供给侧结构性改革。化解稔子坪煤矿煤炭过剩产能39万吨，争取到8家企业列入全区电力直接交易试点，23家企业获得“惠企贷”贷款2.7亿元，5家铁合金企业恢复生产，为企业减少用电、用工、税费等成本超过4.5亿元；支持新市民购买新建普通商品住房补贴、契税补贴等去库存政策释放效应，房地产市场平稳发展。加大力度补齐园区基础设施建设短板，105个重点项目加快建设。深化重点领域各项改革。财税、投融资、金融、医药卫生和商事制度、行政审批制度等改革深入推进，“营改增”全面铺开，减轻企业税负1.91亿元；政府购买服务稳步推广；新农合实现市级统筹，分级诊疗制度加快建设；在全区率先实施“全城通、就近办”和全程电子化企业登记新模式；深化“放管服”改革，清理精简行政审批事项59项，清理规范行政审批中介服务事项112项，开展相对集中行政许可权改革试点。推行灵山县、钦北区镇（街道）权责清单试点，完成全市镇（街道）“四所合一”改革。农村金融改革继续深化，灵山县获批为广西“信用县”。全面推行不动产统一登记制度。党政机关公车改革取得阶段性成果。中马钦州产业园区探索推进法定机构管理模式。

　　（五）着力抓统筹，城乡环境展现可喜变化

　　创建全国文明城市、国家生态园林城市、国家卫生城市、国家食品安全城市“四城联创”深入推进，城市基础设施加快完善。滨海新城“四区三场两街一带一湾”建设加快，茅尾海黄金海岸海滨浴场建成开放，钦州学院实现整体搬迁办学。成功承办第九届广西园林园艺博览会，钦州园博园好评如潮，坭兴陶文化创意产业园建成开园。市政公用设施项目完成投资103亿元，增长41%。国家智慧城市试点取得新成果，信用信息共享平台、电子政务外网工程等投入使用。建成公共自行车服务系统一期，“市民卡”实现南钦北防一卡通。灵山、浦北新型城镇化示范县建设加快推进，浦北县获批为第三批国家新型城镇化综合试点，寨圩、陆屋列入广西百镇建设示范镇。开工建设36个镇级污水处理设施项目，城镇基础设施进一步向农村延伸。狠抓中央环保督察反馈意见整改落实，城区环境空气质量优良率达95.1%。持续抓好生态乡村建设活动，马肚塘村获评“中国美丽休闲乡村”，全市村容村貌大为改观。

　　（六）着力补短板，社会民生持续改善进步

　　坚持共建共享，集中力量解决一批群众关心、社会关注的问题。精准脱贫年度任务全面完成，累计筹措投入资金7.5亿元，市财政专项扶贫资金支出比例排名全区第一，83个贫困村脱贫摘帽、2万贫困人口脱贫。深入实施为民办十件实事工程，投入民生的财政资金达165亿元，占一般公共预算支出82.4%。实施解决主城区“入学难”行动计划，市白石湖实验小学、三十四小学建成使用，高新区实验学校基本建成。实施全民参保计划，全面实行“五险合征”一票征收社保费。全面推行乡村医生签约服务，市直6家、县区14家公立医院和2家民营医院实现新农合即时结报，稳妥有序落实全面两孩政策。继续实施引进领军型创业人才“520”计划，人才工作不断加强。落实就业创业扶持政策，城镇新增就业、农村劳动力转移就业分别完成任务的121.9%、115.5%。农村低保标准由每人每年2700元提到2865元，实现年度低保线与扶贫线两线合一。农村“三留守”人员关爱保护机制进一步完善。农村危房改造力度加大，保障性住房基本建成率、分配入住率分别为197.6%和163.8%。建成北部湾博物馆，获批自治区级非遗项目6项，建成非物质文化遗产传承基地5个，成功举办文化遗产日主题活动、驻港部队“钦州舰”回访等系列重大活动。举（承）办全国中学生足协杯比赛、全国业余乒乓球锦标赛等重大赛事，全民健身活动蓬勃开展。“平安钦州”建设深入推进，社会治安防控体系建设和网格化管理成效明显。强化“一岗双责”，安全生产形势保持平稳。加强食品药品安全综合监管，实现重大食品药品安全零事故。突发事件应急预案体系逐步健全，防汛抗洪、抢险救灾和疫情防控工作有效开展。注重解决群众合理诉求，信访工作取得新成效。加强劳资纠纷调处，促进形成和谐的劳动关系。

　　过去的一年，民族、宗教、外事、侨务、农机、水库移民、供销、新闻出版、广电、统计、贸促、人防、海防、科普、气象、水文、档案、决策咨询、方志、社会科学等工作取得新进展，防震减灾工作再次荣获全国先进，海关、检验检疫、海事、边检、消防、税务、人民银行、银监、供电、烟草、通信、邮政、盐务、各金融保险机构等为我市经济社会发展作出了新贡献，妇女儿童、残疾人、老龄和关心下一代等事业取得新进步，国防教育和后备力量建设深入开展，边海防基础设施建设进一步加强，双拥共建工作取得新成绩，民族团结进步创建活动扎实推进，各民族和睦相处、和衷共济、和谐发展。

　　过去的一年，我们深入开展“两学一做”学习教育，全面贯彻党的十八届六中全会精神，增强“四个意识”特别是核心意识、看齐意识，切实落实中央八项规定精神，不断加强政府自身建设，改进工作作风，提高行政效率。推进依法行政，深化政务公开，完善决策程序，不断健全权力运行制约和监督体系，切实做到有权必有责、用权受监督，政府工作走上法治化、规范化轨道。启用市民服务中心，推进行政审批服务提速，改进和优化公共服务流程，提高公共服务质量和水平。自觉接受市人大及其常委会的法律监督、工作监督和市政协的民主监督，主动听取各民主党派、工商联和社会各界意见建议，人大代表建议和政协提案办结率100%。加强廉政建设、行政监察、审计监督，强化效能督查、政务督查，严肃查处“庸懒散”行为，风清气正的干事氛围更加浓厚。

　　各位代表，2016年是不平凡的一年。在经济下行压力加大、各种困难挑战交织的情况下，取得这些成绩实属不易。这是自治区党委、政府和市委正确领导的结果，是市人大、市政协监督支持的结果，是全市上下团结奋斗、苦干实干的结果。在此，我代表市人民政府，向广大干部群众，向市人大代表、政协委员、各民主党派、工商联、人民团体和离退休老同志，向驻钦人民解放军、武警官兵和中直、区直单位，向参与和支持钦州发展的各界朋友，表示衷心的感谢和崇高的敬意！

　　肯定成绩的同时，我们也清醒看到发展中存在的困难和问题：经济总量偏小、发展基础相对薄弱；产业转型滞后，传统资源型产业比重偏高，新兴产业还没有形成规模，龙头企业不多、带动力不足，发展后劲和创新能力亟待增强；税源结构单一，财政增收乏力，收支矛盾突出等问题仍将持续较长时期；开放平台综合效应未能形成，港口集疏运体系有待完善；城镇功能配套和管理水平亟需提升；基本公共服务等民生改善还须付出更大努力；干部队伍中仍然存在主动作为意识不强、改革创新精神不足和“庸懒散”现象；法治政府建设任重道远，营商环境需进一步优化，等等。对于这些问题，我们绝不回避、绝不遮掩，一定采取有效措施，切实加以解决。

　　二、2017年奋斗目标和工作重点

　　2017年是实施“十三五”规划承上启下的重要一年，是党的十九大召开之年，也是全面贯彻落实自治区第十一次党代会、市第五次党代会精神的第一年，做好今年工作意义重大。面对错综复杂的国内外发展环境，面对繁重艰巨的改革发展任务，我们必须紧抓发展第一要务不放松，主动适应新常态、抢抓新机遇、展现新作为，不辜负400万钦州人民的热切期盼。今年政府工作的总体要求是：全面贯彻党的十八大和十八届三中、四中、五中、六中全会以及中央、全区经济工作会议精神，统筹推进“五位一体”总体布局和协调推进“四个全面”战略布局，紧紧围绕“三大定位”“三大生态”和“两个建成”目标，坚持稳中求进、创新有为，全力实施“创新年”活动，以创新深化开放合作，以创新破解发展难题，以创新激发发展活力，全力稳增长、促改革、调结构、惠民生、防风险，促进经济持续平稳健康发展和社会和谐稳定，为实现“两个建成”目标奠定良好基础，以优异成绩迎接党的十九大胜利召开。

　　今年经济社会发展的主要预期目标是：地区生产总值增长8%，规模以上工业增加值增长10.8%，规模以上工业总产值增长12%，固定资产投资增长13%，财政收入完成154.1亿元，港口吞吐量7300万吨，集装箱吞吐量175万标箱，社会消费品零售总额增长9%，外贸进出口总额增长6%，城镇居民人均可支配收入增长8%，农村居民人均可支配收入增长9.5%，居民消费价格指数涨幅控制在3%左右，万元地区生产总值能耗下降、万元地区生产总值二氧化碳排放降低、主要污染物排放总量控制在自治区下达目标内。

　　完成上述目标任务，要集中力量抓好以下八个方面工作：

　　（一）紧盯结构调整主战场，提升发展质量效益

　　推动临港工业集群延伸发展。着眼发展壮大实体经济，以临港产业为主攻方向，争创产业发展新优势。大力发展千亿元石化产业，依托中石油等龙头企业重点延伸芳烃及下游产业链，促进玉柴、泰兴、天恒等石化企业扩大生产规模，积极申报国家级石化产业基地，推动大型炼化一体化等项目落户，加快华谊煤基多联产一期等项目开工建设，促进玉柴石化30万吨异辛烷等项目建成投产。大力发展千亿元装备制造业，推进中船修船资源整合项目投产，启动造船项目，形成修造船产业体系；以力顺轻型载货汽车升级改造项目投产为突破口，编制汽车产业园区规划及可行性研究，培育发展汽车零部件产业，配套形成汽车产业链；依托集装箱干线港，加快发展海洋工程、集装箱制造、港工机械等临港装备制造产业。大力发展百亿元产业，优化发展lng等能源产业，推动粮油食品加工业规模化、集团化发展，延伸发展造纸下游产业链，加快发展现代物流等百亿元产业。培育发展战略性新兴产业，制定出台相关扶持政策，以电子信息、海洋产业等为突破口，规划建设机器人产业园、云计算产业园、新能源汽车产业园等。大力发展海洋生物、现代海洋渔业等海洋经济，努力把海洋产业打造成为我市经济发展的新引擎和新亮点。

　　推动现代服务业提档升级。深入实施“放心消费”和诚信市场创建活动，继续推进东盟商贸城等专业市场建设，培育限额以上商贸企业，挖掘潜在消费。大力发展电子商务和快递业，抓好全国电子商务进农村示范县试点，扶持灵山县三科电商创业园、浦北县电子商务产业园、淘实惠等电商平台发展。推进全域旅游发展，重点建设钦州历史文化旅游核心区、滨海休闲度假带，支持林湖森林公园、千年古陶城·园博园创建4a级景区，继续完善三娘湾景区配套设施，启动三娘湾创建5a级景区工作。积极发展直接融资，培育金融新型业态，开展外资股权投资企业试点，促进金融业健康平稳发展。规划建设白石湖中央商务区等总部经济集聚区，培育发展总部经济。积极发展大健康产业，培育发展信息服务、科技服务、服务外包等新兴服务业态，推进消费升级。

　　推动创新驱动发展。深入实施创新驱动发展战略，广泛开展大众创业、万众创新，支持重大科学研究、重大科技攻关、重大新产品研发和科技成果转化、重大创新平台建设，认定一批市级工程技术研究中心，鼓励民间资本创办科技企业孵化器，争取引进12项以上科技成果落地转化及产业化。深入实施高新产业培育工程，着力发挥企业创新主体作用，推进小微企业创业创新示范基地建设，支持慧宝源、鑫德利等企业开展科研和技术开发，推进钦州（火龙果）国家农业科技园区、浦北县农业科技园区和电子信息核心产业带建设，加快申报创建国家级高新区，大力发展新经济。

　　（二）突出深化改革主旋律，打开动力转换之门

　　深化供给侧结构性改革。推进钦州港铁合金企业整合重组，争取更多企业享受“惠企贷”政策，力争设立电力直接交易机构，新增一批用电大户纳入自治区电力直接交易试点，建立非国有“僵尸企业”处置机制，支持企业依法破产重整、盘活资产。加大棚户区改造货币化安置力度，出台政策支持农民工、外来务工人员等新市民购房需求，鼓励住房租赁消费，保持房地产市场平稳健康发展。健全完善政府性债务管理体系，组织开展风险企业帮扶行动，切实防范金融风险。打好降本增效“组合拳”，实施有利于降低企业制度性交易成本的政策措施，落实好“营改增”政策；扩大行政事业性收费免征范围，降低企业社保缴费比例；加大“大通关”扶持力度，降低企业物流成本。深入推进农业供给侧结构性改革，把增加绿色优质农产品供给放在突出位置。坚持既补发展硬短板也补制度软短板，加强工业园区基础设施建设，增强教育文化卫生等公共服务能力。

　　深化重点领域改革。深化“放管服”改革，加快设立市行政审批局，进一步清理规范行政审批及中介服务事项，开展一体化网上政务服务，全面推进“双随机、一公开”监管，完成市县权力清单和责任清单“两单融合”工作，推行外商投资负面清单管理模式。深化商事制度改革，推进“多证合一、一照一码”试点，完善个体工商户“两证整合”，加强事中事后监管。推进中马“两国一检”通关新模式，建立健全对接区域通关一体化、全国通关一体化的机制，深入推进国际贸易“单一窗口”建设。积极推动建设北部湾中心海关。深化财税改革，强化预算执行与预算安排挂钩机制。继续推广政府和社会资本合作模式，研究设立政府投资引导基金。深化农村综合改革，基本完成农村土地承包经营权确权颁证，健全农村土地流转服务管理平台，盘活农村“撂荒”土地，促进适度规模经营。全面推进乡村规划建设管理制度改革。实施好新一轮市直国有企业改革，积极推进教育、医药卫生、工业园区、供销合作社、华侨农林场、三娘湾旅游管理区体制机制、小型水利工程管理体制、社会组织管理制度等领域改革。构建“亲”“清”政商关系，促进非公经济加快发展。

　　（三）打好扩大投资主动仗，培育经济新增长点

　　抓好重大项目建设。建立“一个重大项目、一个指挥部、一位联系领导、一个服务班子、一份工作计划”的推进机制，健全项目服务专员、项目进度月报和周报等制度，服务前移、挂图作业、专人跟踪、督查到底，统筹抓好600个总投资1000万元以上项目建设，加快钦州热电厂一期、沿海高速公路改扩建钦州段、南钦铁路电气化改造等项目建设，力促贵港至合浦高速公路等项目竣工，大塘至浦北高速公路等项目开工建设，力争每月都有重大项目开竣工。开展项目建设结算大清理。全年固定资产投资力争突破1000亿元。

　　抓好园区基础设施建设。深入开展园区基础设施三年攻坚活动，以钦州港经济技术开发区、钦州高新区及县域工业园区为重点，推进园区路网、土地平整、供水、污水处理等项目建设，加快市区与中马钦州产业园区、钦州保税港区公共交通一体化建设，开工建设金鼓大街改扩建工程、泉水工业园进园大道工程，争取建成鹰岭作业区至石化产业园公共管廊、钦州高新区二期标准厂房、陆屋临港产业园陆阳新城路网工程二期、皇马工业园路网及供水、排污管网等项目，力争全年园区基础设施建设项目完成投资50亿元以上。

　　抓好要素保障。加大财政收入组织力度，完善国税、地税合作征收管理模式，强化税收征管，培育壮大财源基础，继续盘活财政存量资金，发挥好财政资金的引导作用和乘数效应。创新开发金融产品，开展发行中期票据、短融、企业债，开展金融租赁等融资模式，加大金融支撑项目建设力度，鼓励外商投资，引导社会资本投向重大基础设施、先进制造业、战略性新兴产业、现代服务业、城镇化建设、社会事业、生态环保等领域，开辟投资新领域。推进土地、海域收储，加大已出让土地的清理力度，重点处理已出让地块的遗留问题，增加土地有效供给。

　　（四）实施开放带动主战略，调动一切积极因素

　　全力促进港口开发建设。启动大榄坪区域整体开发和大环作业区建设，加快有色金属物流园、正邦物流、金鼓江作业区14—17#泊位工程及配套仓储等项目建设，力争开工建设20万吨级集装箱航道、钦州港东航道改扩建一期、火车东站至三墩铁路、鹰岭分区车场及鹰岭石化铁路专用线等，实现30万吨级航道和油码头同步运营。推进钦州保税港区“智慧港区”建设，争取新开通至马来西亚等东盟国家港口的集装箱班轮航线，增加开通至西南中南地区铁路集装箱班列、至泰国林查班港的“水果快线”，逐步构建面向东盟、辐射西南中南及国内沿海的区域性国际航运中心。加快鹰岭作业区规范化建设，争取获批活牛指定进口口岸，推进国投煤炭码头、保税港区和三墩岛相关码头获批对外开放。

　　全力发挥钦州平台效应。主动融入“一带一路”建设，创新开展先行先试，复制推广自贸试验区先行先试政策，力争在贸易和投资便利化、金融开放、行政管理等方面取得新突破。积极申报建设自治区级cepa先行先试示范基地。推进中国—东盟港口城市合作网络建设，建立与东盟10国“一国一港”合作机制，建成中国—东盟水上训练基地、中国—东盟海洋气象监测预警中心；精心策划和举办好首届“关丹日”活动，扩大东盟港口城市友好交流圈。推进钦州保税港区封关围网整改验收，加强与马来西亚关丹港通关便利化合作，开展跨境电商网购保税进口业务，扩大肉类、水果进口和汽车进出口等特色业务，建成棉纺产业园标准厂房等项目，发展电子信息等加工贸易产业，培育飞机整机进口贸易交付等通航产业。支持中马钦州产业园区建设中马科技园、智慧产业园等公共服务和产业平台，加快稀土高铁铝合金电缆、鑫德利光电科技产业等项目建设。继续推进钦州港经济技术开发区扩区申报，加快国家进口贸易促进示范区核心区建设，争取获批进口毛燕指定口岸。实施第二轮“加工贸易倍增计划”，促进外贸企稳向好。

　　全力促进精准招商。深入推进招商引资三年行动计划，推进委托招商、以商招商，扩大招商网络和资源，更好地发挥外来投资对促进实体经济发展的作用。突出龙头企业和产业链招商，聚焦石化、装备制造、现代服务业等重点产业和珠三角、东部沿海发达地区等重点区域，力争引进一批石化、装备制造等重大产业链项目，引进培育大数据、电子信息、新能源工业、新材料等产业，全年新引进亿元项目100个以上，引进到位资金527亿元。

　　（五）抓住城乡统筹主线条，推进区域协调发展

　　完善城市基础设施。加快滨海新城建设，开工建设市技工学校、市外国语学校等项目，基本形成白石湖商业圈、大学城创业街、辣椒槌滨海景观带、“欢乐沙井岛”等特色城市功能区。完善“一环二横四纵一联”城市路网，开工建设北部湾大道至中马钦州产业园区道路、钦州湾大道升级改造等工程，建成桅杆坳至清水窝城市道路、钦江五桥等项目，力争打通城市北半环，构建主城区连接中马钦州产业园区的城市大通道。推进滨海新城、中马钦州产业园区地下综合管廊试点项目，加快海绵城市试点。推进市区“断头路”、小街小巷、排水系统等改造建设，畅通城市微循环交通系统。

　　提升城市管理品质。坚持规划引领，开展城市总体规划、土地利用总体规划和海洋功能区划“多规合一”，实施重点区域城市设计，完成高新区等一批控规和专项规划。加快完善教育、医疗、养老、公交等城市公共服务设施布局和建设，推进郁江调水工程、城市供水水源提升工程建设。引入棚户区改造项目加快推进上沙村、沙坪村等城中村改造。深化“园林生活十年计划”，积极参与“美丽广西·宜居城市”建设活动，加快国家生态园林城市建设，推进城市公园、主干道、休闲广场绿化彩化和基础设施提升建设，建设一批城市公园和街道绿化工程，形成钦州特色城市绿化景观。加快智慧城市建设，启动实施公共自行车服务系统二期等项目建设。加大城市管理综合执法力度，深化城市环境综合整治，加强城市内涝治理及市政道路维护管理。

　　推进新型城镇化。加快灵山、浦北、钦北产城互动园区建设，促进新城与园区融合发展，扩大县城产业规模，促进人口集聚。加快灵山、浦北创建国家园林城市步伐，推进广西新型城镇化示范县、百镇示范工程、乡土特色示范村建设，着力培育陆屋、北通、那丽、小董等一批特色小镇。深化户籍制度改革，引导农村人口向县城、镇区集聚，力争常住人口城镇化率达到39%。推进中心城区连接县城、镇村交通大通道建设，争取开工建设浦北张黄至钦州港一级公路，增强辐射带动作用。夯实镇村基础设施，提高村庄规划和民居设计水平，统筹推进城乡规划、产业布局、文化教育、医疗卫生、劳动就业和社会管理一体化发展，加快农村道路、农田水利、给排水、绿化等设施建设，推动城镇市政设施和公共服务向农村延伸。

　　（六）壮大县域经济主力军，挖掘地方发展潜力

　　稳步发展现代农业。严格落实耕地保护制度，突出抓好粮食高产创建和绿色增产模式攻关，确保全年粮食总产量110万吨以上，守住粮食生产能力不降低、农民增收势头不逆转、农村稳定不出问题的三条底线。坚持农民主体地位，继续推进现代特色农业示范区和“双高”糖料蔗基地建设，大力发展秋冬种生产，促进农民增收致富。深入实施特色农业提升工程，加快发展农产品深加工，壮大发展林下经济，促进一二三产业融合发展。加快建设一批农业标准化生产基地，培育一批“三品一标”农产品。立足天然富硒土壤优势，大力发展富硒农业产业，创新推行有机稻、富硒稻和稻田生态立体种养模式。加大现代渔港规划建设，提升“钦州大蚝”品牌影响力。培育新型农业经营主体，争取新认定市级农业产业化重点龙头企业5家以上，新发展农民专业合作社50家、家庭农场50家。

　　壮大发展县域工业。强化质量效益导向，坚持改造提升传统产业和培育壮大新兴产业并重，深入实施县域工业提升工程，完善灵山工业区、浦北县城工业园、钦南金窝工业园、钦北皇马工业园配套设施，集中力量抓好食品加工、电子信息、林木加工、生物医药、新材料等特色产业发展，建设灵山循环经济产业园、浦北风电、钦南康熙岭渔光互补光伏电站、钦北生物医药产业园等一批项目，促进灵山业成化工、浦北凯兴电子、钦南华汇镍业、钦北生物质发电等项目建成投产，形成县域错位发展、多元发展格局，提升县域工业规模、效益和质量。

　　打造县域旅游名片。加快发展乡村旅游、休闲旅游，启动建设钦北鹰山温泉休闲旅游等项目，推进越州天湖休闲农业观光旅游等项目建设，建成北部湾望海岭国际滑翔伞基地，推动八寨沟、五皇山创建国家生态旅游示范区，积极支持钦南区创建国家级全域旅游示范区，争取把六峰山、大朗书院创建成为4a级景区，打造钦北山水生态观光带、灵山浦北民俗风情体验带，形成集休闲、观光、生态、旅游为一体的新型农林渔产业形态和消费业态。提高旅游对经济的贡献率，把旅游打造成经济发展新支柱、富民增收新引擎。

　　主动承接产业转移。实施全域招商，以县区产业园区为载体，更大力度承接国内外产业转移，利用产业特色优势、综合成本优势、资源优势和区位优势，重点布局本地优势资源型、劳动密集型、进口资源加工型、临港配套型等产业，推动产业集群式转移，促进县域经济规模扩张和产业优化。

　　（七）突出绿色发展主色调，营造优越自然生态

　　加强生态系统建设。坚定生态优先理念，协调好环境保护和经济社会发展的关系，营造山清水秀的自然生态。深入实施绿色发展战略，加强生态功能区和生态带建设，加强饮用水水源地保护，逐步建立生态补偿机制，科学有序开发各类资源。保护和修复沿海红树林海洋生态系统，建设海洋生态林带和海洋生态监控区，进一步增强保护海洋环境的全民意识，共同呵护白海豚赖以生存的美丽家园。加快建设节水型城市，加强工业再生水回用，建立生活垃圾分类回收和再生资源回收体系，基本形成覆盖全社会的资源循环利用体系。倡导绿色发展方式和生活方式，推进公共机构节能，建设一批低碳社区。加大植树造林力度，加强水源林的保护和管理，加快建设国家森林城市。全面推进宜居乡村、生态城镇建设，贯彻落实《广西壮族自治区乡村清洁条例》，继续实施清洁田园“十大工程”，开展农村垃圾治理攻坚、乡村水环境综合整治，健全乡村保洁机制，打造绿色环境、绿色建筑、绿色生产、绿色生活，让人民群众享有更健康舒适的环境。

　　狠抓环境综合整治。严格执行“水十条”“大气十条”和“土十条”，强化约束性指标管理，控制主要污染物排放总量。强化工业废气、粉尘扬尘、建筑渣土和汽车尾气污染治理。全面推行市、县、镇三级“河长制”，实行最严格水资源管理制度，推进河流湖库综合整治，系统开展钦江、茅岭江、大风江等重点流域及黑臭水体治理。科学划定海洋生态红线，加强核心海岸线资源保护，强化近岸海域等综合治理，实施茅尾海、三娘湾、钦州港陆域入海污染整治工程及海域环境综合整治工程，建设蓝色海湾综合整治示范区。深入开展重金属污染防治，加强土壤污染监测和治理，强化农业面源污染防治和畜禽养殖污染防治，加快建设镇级污水处理设施，深入推进农村环境综合整治。完善生态环境动态监测网络，强化环境保护执法监察，依法严惩环境违法行为。继续推进中央环保督察反馈意见整改落实工作，落实环境保护“党政同责”“一岗双责”，强化追责问责，确保钦州生态环境名片不变质、更出色。

　　大力发展生态经济。按照减量化、再利用、资源化的原则，大力发展循环经济，加快石化产业园循环化改造，启动建设循环经济示范岛。发展现代生态农业，推进畜禽规模养殖场生态化改造，推广清洁生产“十大技术”，扎实推进农业循环经济，培育生态农业龙头企业，提升农产品质量安全水平。做强做优生态旅游产业，培育壮大节能环保产业，加快推动建筑节能改造，推广太阳能光电建筑一体化、空气能等可再生能源技术，培育发展一批循环经济示范企业。

　　（八）稳住保障民生主基调，营造良好社会生态

　　全力推进精准脱贫。坚决把脱贫攻坚作为最大的政治责任、最大的民生工程、最大的发展机遇来落实，因户施策、因人施策，进一步压实责任、落实政策、动员群众。继续实施脱贫攻坚十大行动，把产业扶贫摆在第一位，开展产业扶贫示范小区带动扶贫脱贫试点，引导民营企业发挥行业特色优势参与扶贫攻坚，加快实施易地扶贫搬迁，鼓励群众积极参与、不等不靠、自力更生、勤劳致富，帮助贫困村、贫困户增强自我“造血”机能，促进扶贫开发与做大做强县域经济良性互动。加强扶贫工作队员业务培训，提升精准帮扶能力。创新完善财政专项扶贫资金分配使用监督管理机制，建立常态化巡查督导问责机制，建好扶贫资金的“防护墙”，防止弄虚作假、数字脱贫。年内实现80个贫困村、3.65万贫困人口拔穷根、挪穷窝、摘穷帽。

　　提升社会保障水平。加快众创空间、农民工创业园等建设，推进创业担保贷款工作，激发大众创业热情。全面治理拖欠农民工工资违法行为，进一步构建和谐劳动关系。健全职业技能培训制度，抓好重点群体就业，支持返乡农民工、大学生、失地农民、失海渔民和农村留守人员创业创新，扎实开展未就业毕业生实名制服务，推进订单式培训和企业新型学徒制试点，加强对灵活就业、新就业形态的扶持，提供更高质量就业创业服务，让广大群众都有事干、有钱挣、有奔头。实施统一的城乡居民医保制度，统筹推进城乡社会保障体系建设，推进养老保险扩面征缴，重点抓好非公有制企业参保，提升全民参保率。完善社会救助体系，健全与社会保障制度相衔接的特困人员救助供养机制。继续抓好养老服务机构公建民营、信息化建设、标准化建设、医养融合发展、人才培养等工作，加快社会养老、社会福利和慈善事业发展。继续做好保障性住房及农村危房改造工作，让群众住有所居。

　　积极创新社会治理。严格落实安全生产工作责任制，加快建立隐患排查治理体系和风险防控体系，深化安全隐患排查治理和专项整治，坚决防范重特大事故。用最严谨的标准、最严格的监管、最严厉的处罚、最严肃的问责，加强食品药品安全监管，确保人民群众饮食、用药安全放心。坚持以人民为中心创新社会治理，全面落实社会治安综合治理领导责任制，开展“综治中心建设年”活动，强化社会网格化管理，推进社会治安防控体系建设，依法严厉打击违法犯罪活动，扎实开展反恐防控工作，提升群众的安全感和满意度。依法规范互联网管理，净化网络环境。实行网上受理信访制度，持续推动信访“三无”县（区）创建活动，综合施策解决群众合理诉求。推进社会诚信体系建设，营造诚信社会环境。全面提升防灾减灾救灾能力，做好地震、气象、水文监测预报和地质灾害预警防治及防汛抗旱等工作，进一步加强应急救援能力建设，努力创建全国防震减灾安全示范城市。

　　大力发展社会事业。加大对农村、山区学校以及薄弱环节、重点领域的支持力度，全力推进县域义务教育均衡发展，加快解决城区中小学“大班额”问题。大力发展职业教育、民办教育、学前教育和特殊教育。完成北部湾大学二期建设。抓好卫生与健康工作，推进城市公立医院改革，加快组建医疗联合体，深入实施分级诊疗制度，落实基层医疗卫生机构能力建设行动计划，构建钦州“健康云”平台，提升计划生育服务管理水平，增强重大疾病防控能力。大力培育和弘扬社会主义核心价值观，持续深入开展全国文明城市、国家卫生城市、国家食品安全城市创建活动。扎实推进现代公共文化服务体系建设。启动申报中国历史文化名城创建工作，继续推进坭兴陶烧制技艺申报联合国教科文组织人类非物质文化遗产名录工作。加大文物保护力度，深入推进“扫黄打非”。支持浦北县申报“世界长寿之乡”。举办世界沙排巡回赛、第六届市运会等重大体育赛事，承办好环广西公路自行车世界巡回赛（钦州段），广泛开展群众体育健身活动，促进全民健身与全民健康深度融合。加强国防动员和后备力量建设，加大双拥创建力度，推动军民融合发展。依法保障公民特别是妇女、未成年人和残疾人的合法权益。充分发挥工会、共青团、妇联等群团组织的作用。继续做好新闻出版、档案、社会科学、新型智库、方志、文史、海防、人防、打私、民族、宗教等工作。

　　悠悠万事，民生为重！我们要带着深厚的感情去倾听群众呼声，了解群众疾苦，办好为民实事，让人民群众的生活一年更比一年好，幸福感一年更比一年强！

　　三、加强政府自身建设

　　重任千钧惟担当。面对新形势新任务，牢固树立“四个意识”，坚持依法行政、高效施政、廉洁从政，创新政府管理，优化政府服务，提高政府执行力、公信力，营造风清气正的政治生态。

　　打造法治政府。坚持“法无授权不可为、法定职责必须为”，推进政府机构、职能、权限、程序、责任法定化，完善依法行政制度体系，确保政府活动全面纳入法治轨道。继续健全决策机制，坚持重大行政决策集体讨论决定，加强重大决策第三方评估，严格行政决策责任追究，提高行政决策科学化、民主化、法治化水平。推动决策、执行、管理、服务和结果公开，提升政府透明度。推进行政执法和刑事司法衔接，加强行政复议和行政应诉工作，加大普法力度，健全公共法律服务体系。自觉接受人大及其常委会的法律监督、工作监督和政协的民主监督，高度重视社会公众监督和舆论监督，加强行政监察和审计监督，认真办理好人大代表议案、建议和政协提案。

　　打造有为政府。各级领导干部要把促改革和抓落实放在更加突出的位置，亲自抓、带头干，做到重要改革亲自部署、重大方案亲自把关、关键环节亲自协调、落实情况亲自督察，扑下身子，狠抓落实。要旗帜鲜明为敢于担当的干部担当，为敢于负责的干部负责，给改革创新者撑腰鼓劲，倡导敢干事、能干事、干成事的氛围，建立宽容过错、允许失败的干事创业容错机制，营造敢于担当、主动作为的政务环境，以服务创新、工作创新疏解体制机制痛点堵点，加快实现新旧动能转换，破解制约改革发展的瓶颈和难题。健全并严格执行工作责任制，确保各项政策和任务不折不扣落到实处。强化政府绩效评估和行政问责，坚决纠正不作为、慢作为、乱作为，坚决惩处失职、渎职，重点整治“庸懒散”、为官不为等现象，着力解决缺位、越位、不到位的问题。

　　打造高效政府。以敬民之心行简政之道，多施便民之策，多办利民之事，重视调查研究，坚持眼睛向下、脚步向下，了解基层群众所思、所想、所盼，推进“多评合一、多图联审、联合验收”，实施“双告知、双反馈”制度，最大限度减少审批事项，简化审批环节，缩短审批时限，提高审批效率。利用“互联网+行政审批”推进网上审批，逐渐做到“网上审批为常态、网下审批为例外”。深入推进政务公开，实施“互联网+政务服务”，提高政务服务水平。建立健全各级政务服务体系，推动政务服务向基层延伸，打通服务群众“最后一公里”。

　　打造清廉政府。严格落实中央八项规定精神、国务院“约法三章”，巩固拓展党的群众路线教育实践活动、“三严三实”专题教育和“两学一做”学习教育成果。落实全面从严治党主体责任和监督责任，严格履行“一岗双责”，认真执行《关于新形势下党内政治生活的若干准则》《中国共产党党内监督条例》等规定，把政府系统党风廉政建设和反腐败工作推向深入。深化领导干部经济责任审计，加强重点领域特别是对扶贫项目和资金等落实情况的监察审计，完善土地出让、工程建设、产权交易、政府采购等制度，加强公共资金、公共资源、国有资产监管，推行政府权力清单制度，坚决消除权力设租寻租空间，健全惩治和预防腐败体系，严肃查处违纪违法案件，确保干部清正、政府清廉、政治清明。

　　各位代表，在新一轮竞争中赶超跨越，需要我们心齐劲足、发奋图强。让我们更加紧密地团结在以习近平同志为核心的党中央周围，在自治区党委、政府和市委的坚强领导下，迎难而上，顺势而为，创新实干，为加快实现“两个建成”宏伟目标而努力奋斗！
